# Adapting the DeepSARM approach for dual-target ligand design

**DOI:** 10.1007/s10822-021-00379-5

**Published:** 2021-03-13

**Authors:** Atsushi Yoshimori, Huabin Hu, Jürgen Bajorath

**Affiliations:** 1 Institute for Theoretical Medicine, Inc., 26-1 Muraoka-Higashi 2-chome, Fujisawa, Kanagawa 251-0012 Japan; 2 Department of Life Science Informatics, B-IT, LIMES Program Unit Chemical Biology and Medicinal Chemistry, Rheinische Friedrich-Wilhelms-Universität, Friedrich-Hirzebruch-Allee 6, 53115 Bonn, Germany

**Keywords:** Structure–activity relationships, SAR matrix, Molecular grid maps, Deep generative modeling, Dual-target compound design

## Abstract

The structure–activity relationship (SAR) matrix (SARM) methodology and data structure was originally developed to extract structurally related compound series from data sets of any composition, organize these series in matrices reminiscent of R-group tables, and visualize SAR patterns. The SARM approach combines the identification of structural relationships between series of active compounds with analog design, which is facilitated by systematically exploring combinations of core structures and substituents that have not been synthesized. The SARM methodology was extended through the introduction of DeepSARM, which added deep learning and generative modeling to target-based analog design by taking compound information from related targets into account to further increase structural novelty. Herein, we present the foundations of the SARM methodology and discuss how DeepSARM modeling can be adapted for the design of compounds with dual-target activity. Generating dual-target compounds represents an equally attractive and challenging task for polypharmacology-oriented drug discovery. The DeepSARM-based approach is illustrated using a computational proof-of-concept application focusing on the design of candidate inhibitors for two prominent anti-cancer targets.

## Systematic analysis and visualization of structure–activity relationships

The availability of increasingly large sets of active compounds for many pharmaceutical targets has triggered interest in developing new computational approaches to systematically explore structure–activity relationships (SARs) in such data sets and visualize SARs [1]. Relevant methods include, for example, numerical SAR analysis functions [2, 3], statistical methods to monitor SAR progression in evolving data sets [4–6], and various approaches for SAR visualization. These include the use of scaffold hierarchies [7–9], molecular networks [10, 11], or different views of activity landscapes [12, 13]. However, methods that combine systematic SAR analysis, visualization, and compound design are rare [8, 14]. In this context, the SAR matrix (SARM) approach was developed.

## SAR matrix

**Fig. 1:**
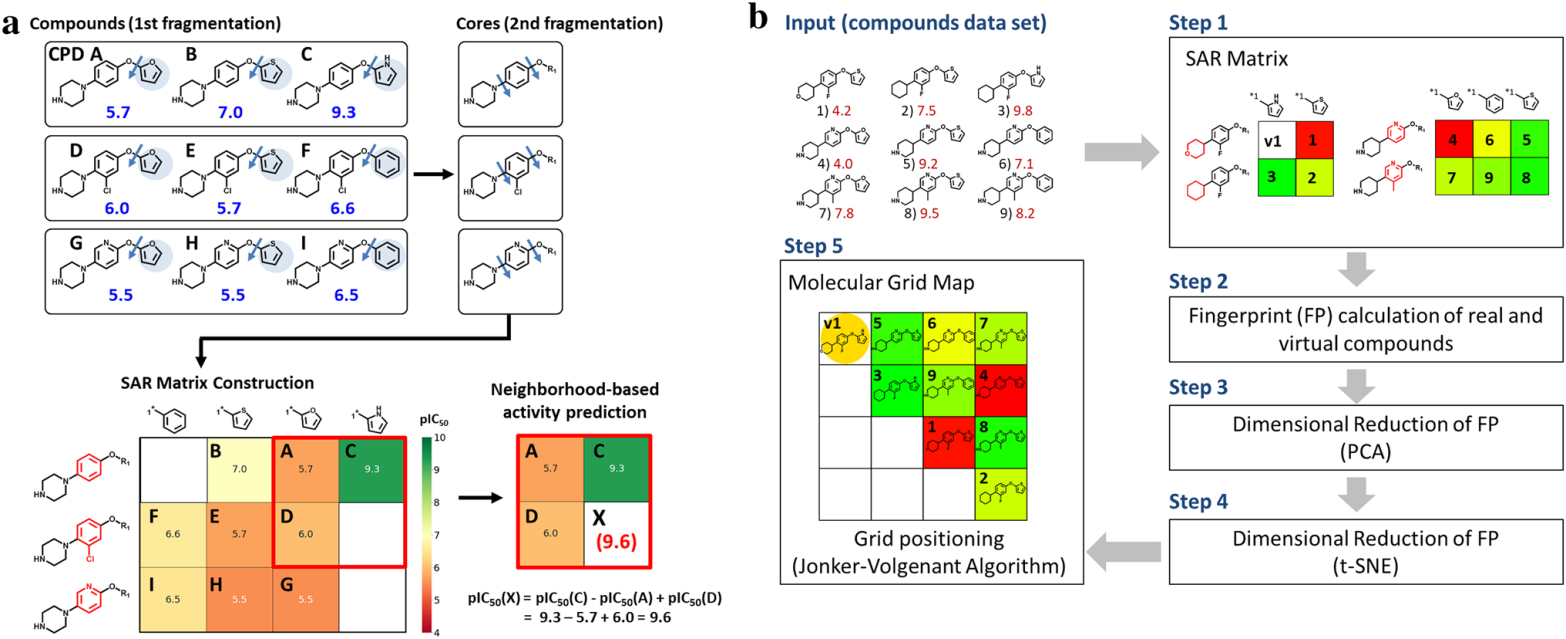

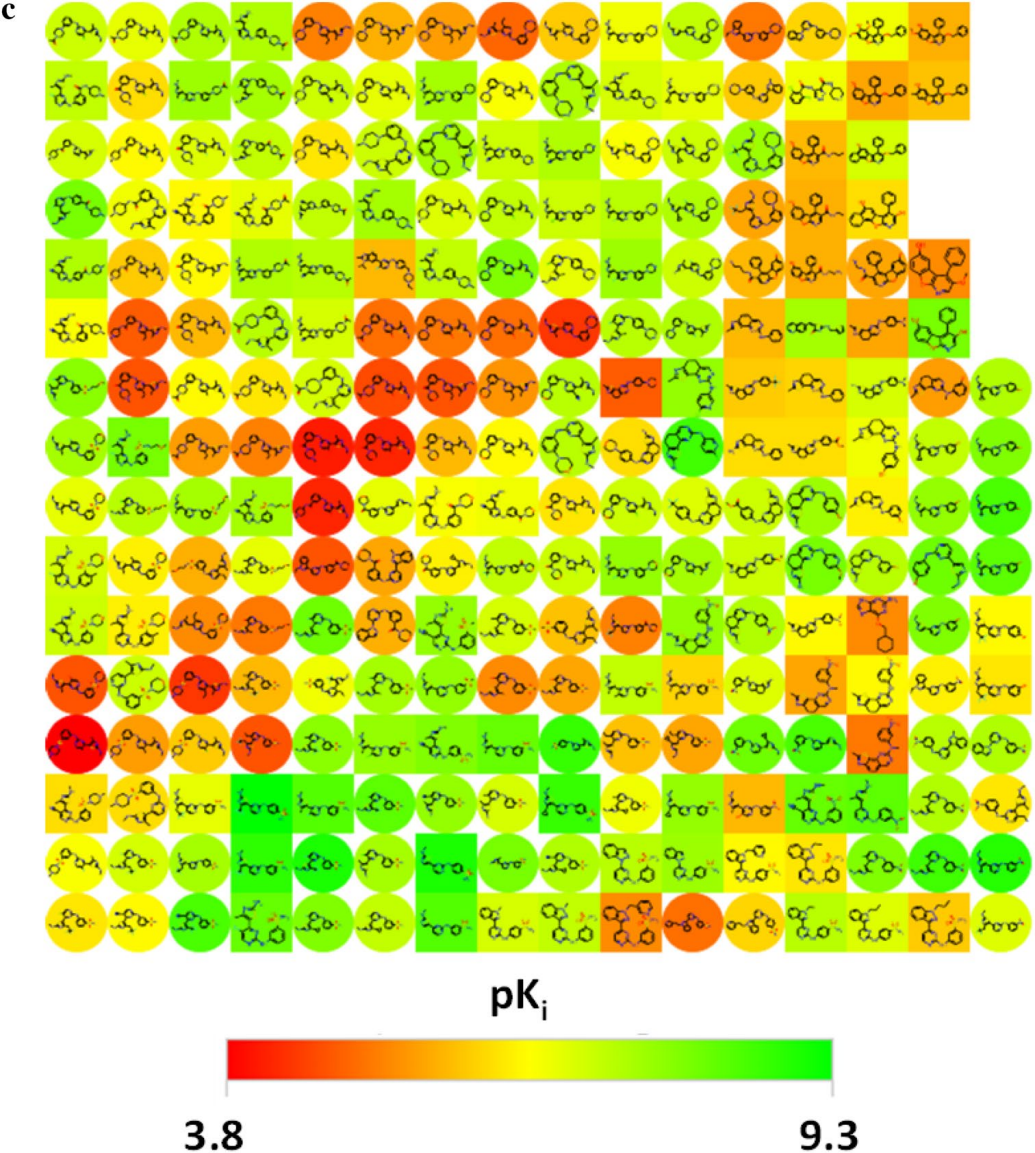
SARM and MGM. **a** SARM construction is illustrated using a model data set comprising nine compounds (CPD A–I; pIC_50_ values are reported in dark blue). Substituents distinguishing analogs are shown on a light blue background. SARM generation is based upon a dual-step fragmentation scheme that identifies analog series with structurally related cores. Substructures distinguishing cores are shown in red. Each SARM cell color-coded by potency represents a unique compound (A–I) and an empty cell a virtual analog, i.e., a not yet explored combination of a core (key) and substituent (value). The potency of a virtual analog (X) is predicted on the basis of suitable compound neighborhoods using local (Free-Wilson-type) QSAR models (lower right). The figure has been taken from ref. [25]. **b** MGM generation is illustrated using another model data set with nine compounds (CPD A–I). Initially, SARMs are constructed (V1 is a virtual analog). Then, similarity calculations are carried out, dimensionality reduction is performed (*PCA* principal component analysis; *t-SNE* t-stochastic neighborhood embedding), and the initial grid positioning of compounds is optimized. The figure was taken from ref. [18]. **c** Shown is an exemplary small MGM for a set of 92 cyclin-dependent kinase 1/cyclin B1 inhibitors (shown on squares) and 156 virtual analogs originating from SARM analysis (circles). Background squares and circles are color-coded by experimental potencies and values predicted using local (Free-Wilson-type) QSAR models, respectively (figure taken from ref. [18])

The SARM methodology and data structure [14] was originally designed to systematically extract analog series with single substitution sites from compound data sets, identify series with structurally analogous cores, and organize these series in a matrix format reminiscent of R-group tables (this matrix is also referred to as a SARM). Thereby, structural relationships in compound data sets are systematically explored. Depending of the nature and extent of available structural relationships, data sets typically yield multiple SARMs, each of which organizes a set of analog series with structurally closely related cores. SARM generation is based upon a dual-step compound fragmentation scheme adapted from matched molecular pair (MMP) analysis [15]. An MMP is defined as a pair of compounds that are only distinguished by a chemical modification at a single site [15]. In the first step, database compounds are subjected to systematic fragmentation of exocyclic single bonds, yielding keys (core structures) and values (substituents), which are stored in an index table. In the second step, the obtained cores are re-submitted to the same fragmentation protocol to identify cores that are only distinguished by a chemical change at a single site (structurally analogous cores), yielding a second index table. Each subset of structurally analogous cores and the compounds containing these cores yield an individual SARM. In this data structure, each row contains an analog series (compounds sharing the same core) and each column compounds from different series sharing the same substituent. Accordingly, the SARM consists of cells that represent all possible combinations of cores and substituents from the subset of related analog series. Each cell represents an individual key-value combination (compound). Hence, cells may contain existing compounds or virtual analogs (i.e., unexplored core and substituent combinations). Therefore, as a desired product of systematic fragmentation and structural organization, SARMs provide virtual candidates that complement and further extend currently available analog space. Hence, the SARM method and data structure integrates structural analysis with compound design. Cells containing existing compounds can be color-coded by potency values, thereby facilitating SAR visualization. Furthermore, the potency of virtual candidates can be predicted on the basis of SARMs using local quantitative SAR (QSAR) models [16] following Free-Wilson additivity principles [17]. Therefore, matrix neighborhoods formed by virtual candidates and experimental analogs with corresponding cores or substituents are identified. For compound potency prediction across different SARMs, machine learning models can also be derived. Figure 1a illustrates the generation of SARMs and their information content.

The global distribution of existing and virtual compounds across SARMs can also be visualized in a meta data structure termed Molecular Grid Map (MGM) [18]. Here, pairwise molecular fingerprint similarity between all SARM compounds is calculated as a reference frame for combining related and unrelated analog series from different SARMs. From the resulting fingerprint space, a 2D projection is generated through dimensionality reduction. Compound positions are then algorithmically mapped to a regularly spaced grid and the positioning is subjected to combinatorial optimization [19] to arrive at a final similarity-based organization and color-coded display of the entire compound population from a set of SARMs. Figure 1b summarizes MGM generation and Fig. 1c shows a representative example. The MGM data structure makes it possible to view all relationships between existing and virtual compounds from SARMs and focus on regions that are rich in SAR information or regions where potent compounds are consistently predicted. SARM and MGM analysis have been successfully applied to identify new active compounds for different targets [20, 21].

Virtual analogs from SARMs result from the recombination of core structures and substituents extracted from existing analog series. Although these virtual candidates further extend analog space for a collection of active compounds, they do not contain novel structural fragments. Accordingly, this close-in compound design strategy is tailored towards hit expansion and lead optimization. Structural novelty of virtual analogs can be further increased by adding novel fragments from external compounds to the design pool. This can be accomplished, for example, through generative modeling using deep learning architectures [22, 23]. Therefore, the DeepSARM approach has been introduced [24]. Generative molecular design using DeepSARM leads to an expansion of SARMs for a given data set through the incorporation of fragments derived from compounds that are active against related targets. Thereby, the number of virtual analogs contained in SARMs further increases. For example, if one is interested in inhibitors of a particular protein kinase, a deep generative model can be derived on the basis of compounds with activity against related kinases (such as the family to which the kinase of interest belongs). Once derived, the model is fine-tuned for the primary kinase target by focusing on its known inhibitors. For SARM expansion with novel virtual analogs, DeepSARM employs a recurrent neural network structure [24] that is discussed below. Details of the SARM approach and its DeepSARM extension have recently been reviewed [25].

## DeepSARM architecture

**Fig. 2:**
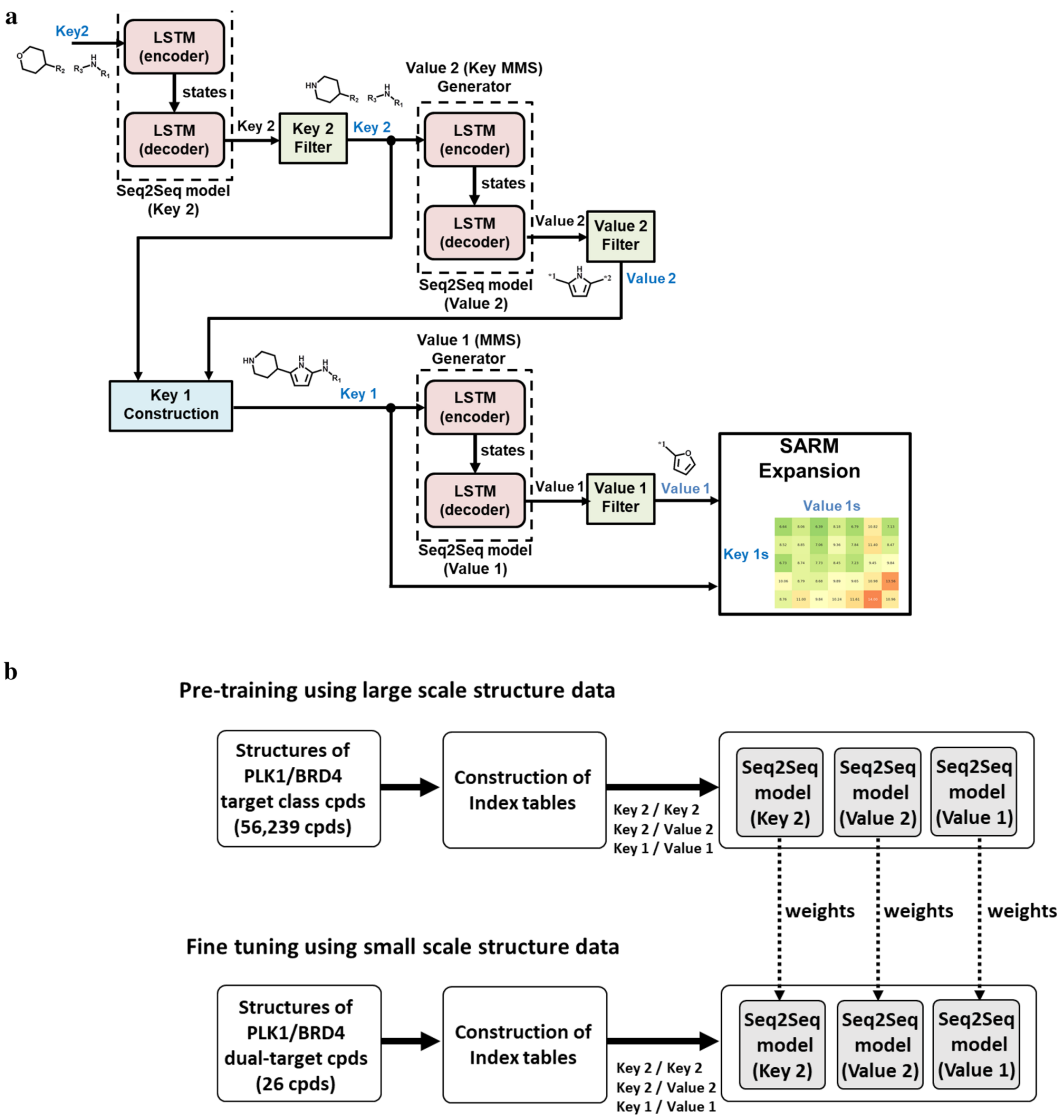
DeepSARM. **a** The central DeepSARM recurrent neural network architecture is outlined, as described in the text (figure taken from ref. [25]). **b** DeepSARM model derivation including the pre-training and fine-tuning steps is summarized. “cpds” stands for compounds (figure adapted from ref. [24] and modified)

The DeepSARM recurrent neural network structure depicted in Fig. 2a includes three encoder-decoder generator components [26, 27], each of which consists of two long short-term memory (LSTM) units [28]. An encoder-decoder generator derives sequence-to-sequence (Seq2Seq) models for transforming one data sequence into another. For generative modeling, key and value fragments are represented as SMILES strings [29] that are vectorized [26]. The Seq2Seq models were generated with Keras [30]. Calculation parameters and further calculation details were reported previously [24].

Key and value fragments 1 and 2 originate from the first (compound) and second (core) fragmentation step and the corresponding index tables. The DeepSARM key 2 generator (first Seq2Seq model) learns to construct new key 2 structures from input key 2 fragments. In the second phase, the value 2 generator (second Seq2Seq model) derives new value 2 fragments from the key 2 structures obtained in the previous step. The resulting key 2 and value 2 fragments yield new key 1 fragments. In the third phase, the value 1 generator (third Seq2Seq model) uses these key 1 fragments as input to produce new value 1 fragments. Newly derived key 1 and value 1 fragments expand original SARMs with new virtual compounds (key-value combinations). Filters between Seq2Seq models rank fragments on the basis of log_likelihood scores derived from the probability distribution of the decoder.

Figure 2b summarizes the derivation of DeepSARM models. The Seq2Seq model components are initially trained with a large number of compounds with activity against a target family or group (or combinations of families or groups, as further discussed below). During the first training phase, the recurrent neural network learns both the SMILES syntax and the structural spectrum of the source compounds. Hence, typically large numbers of compounds with desired structure–activity relationships are initially used. During the second training phase, the resulting model is fine-tuned focusing on compounds with activity against an individual target of interest (e.g., a member of the target family or group used for pre-training). This process leads to the adjustment of initially derived transferred model weights.

Through this pre-training and fine-tuning procedure, key and value fragments that are not contained in compounds active against the primary target, but are related to them on the basis of log_likelihood scores from Seq2Seq models, enter SARM design. New key and value fragments meeting a pre-defined log_likelihood criterion are then added to the respective SARM(s) on the vertical and horizontal axis, respectively. Their combinations give rise to new virtual analogs (key–value combinations), leading to SARM expansion. The log_likelihood score of a new virtual analog is obtained as the sum of the individual scores of its fragments and may be used to prioritize virtual candidates.

## DeepSARM concept for dual-target ligand design

We have reasoned that the DeepSARM approach might be further extended for the computational design of compounds with desired activity against two different targets (dual-target ligands). Such ligands are prime candidates for polypharmacology [31, 32], an increasingly popular therapeutic approach in drug discovery. Polypharmacology refers to the concomitant engagement of multiple targets and the ensuing pharmacological effects through the administration of compound combinations or multi-target ligands [31]. Such multi-target engagement is often critical for achieving therapeutic efficacy in areas such as oncology or neurodegenerative diseases [31, 32]. Polypharmacological agents have often been serendipitously discovered. Accordingly, the design of dual-target ligands with pre-defined activity has become a hot topic in drug discovery [32]. DeepSARM can be adapted for the rational design of dual-target ligands, as discussed in the following.

The two-phase training procedure of DeepSARM outlined above was originally conceived to enrich extrapolative compound design for a specific target with structural information from compounds active against related targets. For example, a model might initially be pre-trained for a large kinase group and then fine-tuned for an individual member of this kinase group. This learning strategy is in principle transferable to dual-target compound design. Generative DeepSARM modeling is principally focused on expanding SARMs with novel analogs, which further expands bioactive chemical space surrounding active compound series. By combining chemical space for different targets and corresponding target classes, dual-target ligand design becomes feasible using the DeepSARM framework.

Specifically, if we aim to generate dual-target ligands with activity against target A + B, then a DeepSARM model can initially be trained with active compounds available for target class A (i.e., the class to which target A belongs) plus active compounds available for target class B. Hence, instead of an individual target class, a compound pool resulting from the combination of two classes is used. This is followed by fine-tuning, for which at least two different strategies can be considered. Ideally, if at least small numbers of dual-target ligands shared by target A and B are available, these compounds can be directly used for fine tuning, aiming to generate additional dual-target ligands with novel structural features derived from the compound pool. Alternatively, if no dual-target ligands are known, fine-tuning can be attempted on the basis of combined compounds active against target A or target B. This strategy aims to identify dual-target ligands that combine structural features from these active compounds taking target class information into account. Depending on the nature of the target combinations of interest and the compound data available, additional fine-tuning strategies might be envisioned by varying the compositions of compound sets for learning.

## Computational proof-of-concept application

### Target combination and data

As an exemplary proof-of-concept application, dual-target ligand design focusing on serine/threonine polo-like kinase 1 (PLK1) [33] and bromodomain-containing protein 4 (BRD4) [34] is reported. PLK1 is a central regulator of cell cycle progression and DNA damage responses. BRD4 is a chromatin-targeting protein that recognizes acetylated lysine residues and acts as an epigenetic regulator. Uncontrolled PLK1 and BRD4 activities are implicated in carcinogenesis. Accordingly, both proteins are intensely investigated as anti-cancer targets [33, 34] and represent an attractive target combination for polypharmacology.

From ChEMBL (version 27) [35], 309 and 1340 inhibitors with reliable activity measurements were obtained for PLK1 and BRD4, respectively, 26 of which were found to be active against both targets (known dual-target compounds). PLK1 and BRD4 were assigned to the “protein kinase” and “bromodomain” target class, respectively, following the ChEMBL classification scheme [35]. For the combined target classes, a total 56,239 compounds with reliable activity data were obtained (including PLK1 and BRD4 inhibitors).

### DeepSARM design

First, we subjected the combined 1649 PLK1 and BRD4 inhibitors to SARM analysis. Then, for dual-target ligand design with DeepSARM, the following strategy was applied in this case: For initial training, the combined 56,239 target class compounds were used. Fine-tuning of the resulting model was then carried out with the set 26 known PLK1/BRD4 dual-target inhibitors. The training strategy is summarized on the left in Fig. 2b. Since a small set of known dual-target compounds was available in this case, preference was given to these compounds over individual PLK1 and BRD4 inhibitors (which were included in the initial training phase). Thereby, fine-tuning capacity on the basis of small compound sets with dual-target activity was assessed.

**Fig. 3:**
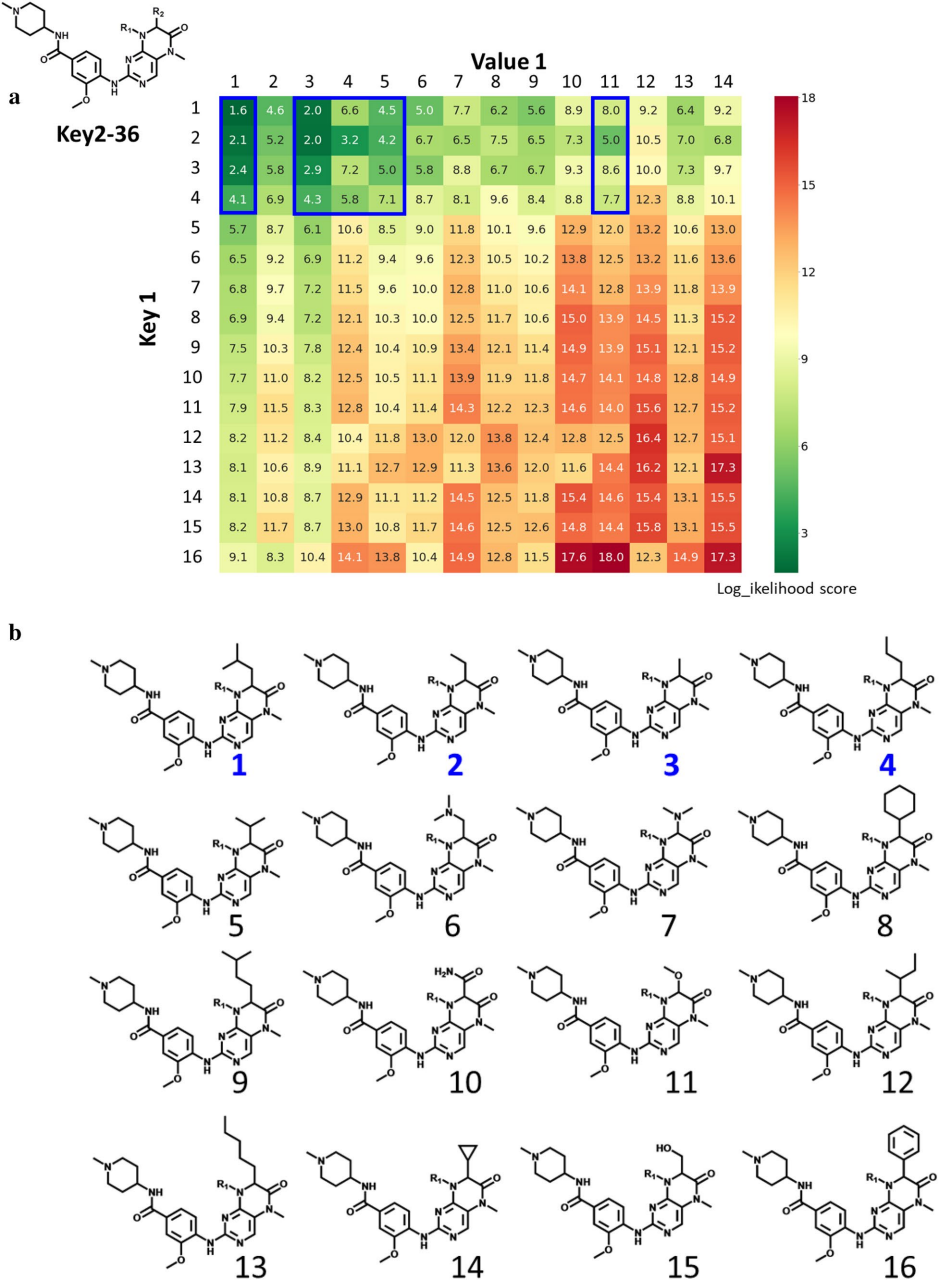

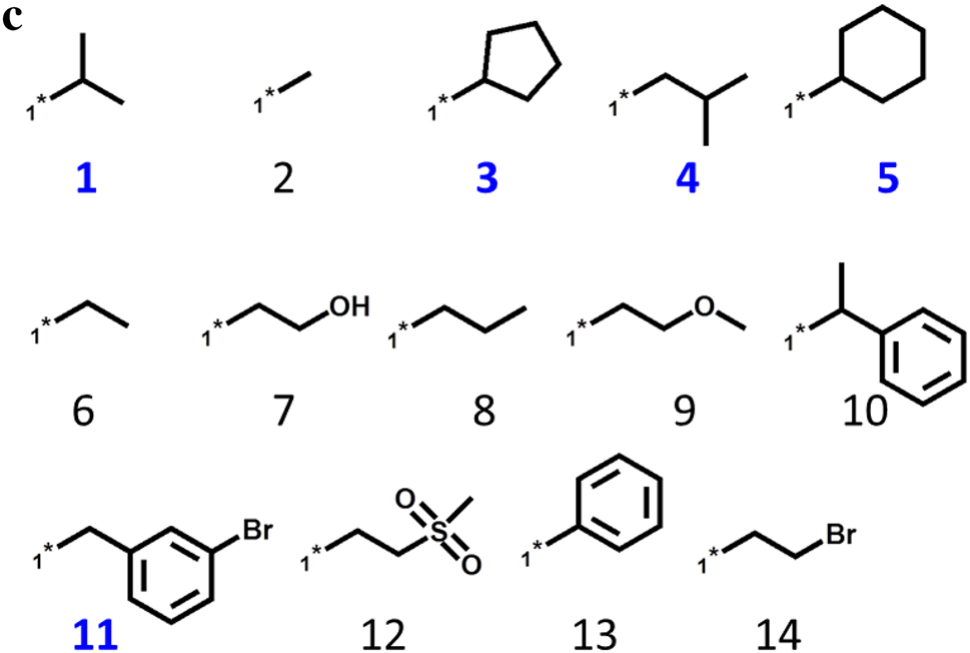
SARM expansion. In **a**, an exemplary SARM is shown resulting from the analysis of PLK1 and BRD4 inhibitors using DeepSARM and Key2-36 as input. Compounds forming the original SARM (framed in blue) resulted from combinations of keys 1–4 and values 1, 3–5, and 11. All other keys and values originated from generative modeling, which further expanded the SARM with more than 200 new virtual compounds. Compound cells are color-coded according to log_likelihood scores originating from DeepSARM using a continuous color spectrum ranging from green (favorable) over yellow to red (unfavorable). In **b** and **c**, keys and values comprising the SARM are shown, respectively, including fragments derived from known inhibitors forming the original SARM (blue numbers) and others generated by DeepSARM (black numbers)

Known PLK1/BRD4 dual-target compounds were found in 86 original SARMs, all of which were expanded using generative DeepSARM model. Figure 3a shows an exemplary SARM expansion generated on the basis of Key2-36 from original SARMs following DeepSARM’s sequential generative modeling protocol (Fig. 2a). The original SARM consisted of four keys (1–4 in Fig. 3b) and five values (1, 3–5, and 11 in Fig. 3c), which represented10 dual-target ligands10 virtual analogs. The expanded SARM contained 16 keys and 14 values and 204 new virtual analogs. The 12 keys and nine values originating from DeepSARM are depicted in Fig. 3b and c, respectively. Compound cells in the expanded SARM are color-coded on the basis of log_likelihood scores. Decreasing scores indicate increasing compound probabilities assigned by DeepSARM, which reproduced existing compounds with low scores. The expanded SARM in Fig. 3a represents a typical DeepSARM result.

### Prioritization of candidate compounds

**Fig. 4:**
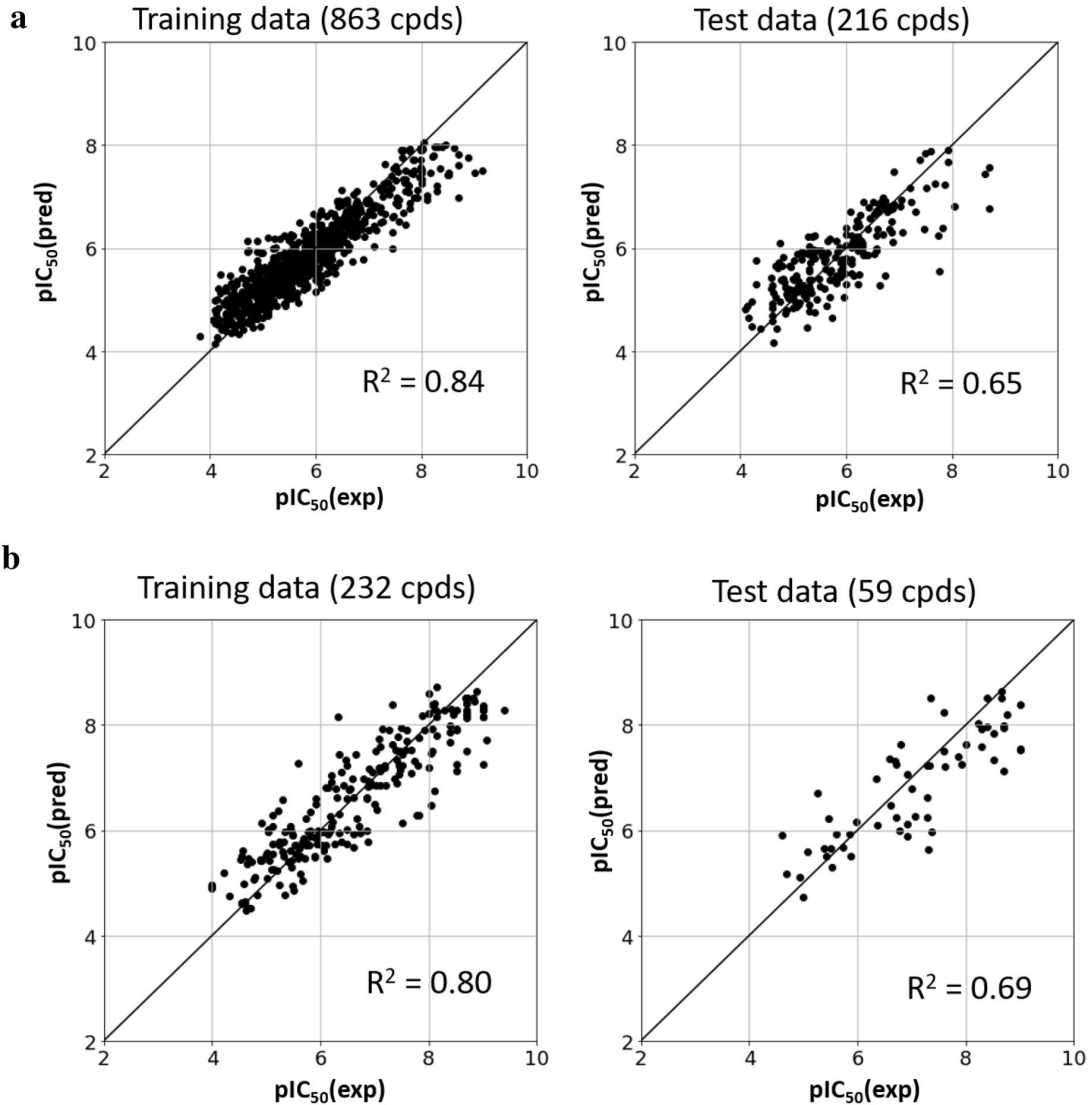
Activity prediction models. Shown are representative results of machine learning models derived to predict the potency values of **a** BRD4 and **b** PLK1 inhibitors. For training and test compounds, comparisons of experimental and predicted potency values are reported

To aid in compound prioritization, PLK1 and BRD4 inhibitor activity prediction models were derived using LightGBM, an implementation of the gradient boosting decision tree algorithm [36]. The goal was to identify virtual candidates for which high potency values were predicted for both BRD4 and PLK1, serving as an indicator of dual-target inhibitor potential. For model building, 1079 BRD4 and 291 PLK1 inhibitors were selected, for which IC_50_ measurements were available. For the remaining small subsets of inhibitors, only K_i_ values were available. Since IC_50_ and K_i_ measurements cannot be directly compared, the latter values were not considered for modeling in this case. In model construction, 80% of the compounds were used for training and 20% for testing. Figure 4a and b show the results of representative potency predictions for BRD4 and PLK1 inhibitors, respectively. In both cases, reasonable prediction models were obtained with R^2^ values for training and test set predictions of ~ 0.8 and ~ 0.7, respectively. For most test compounds, potency was predicted within an order of magnitude, which was sufficiently accurate for compound prioritization.

**Fig. 5:**
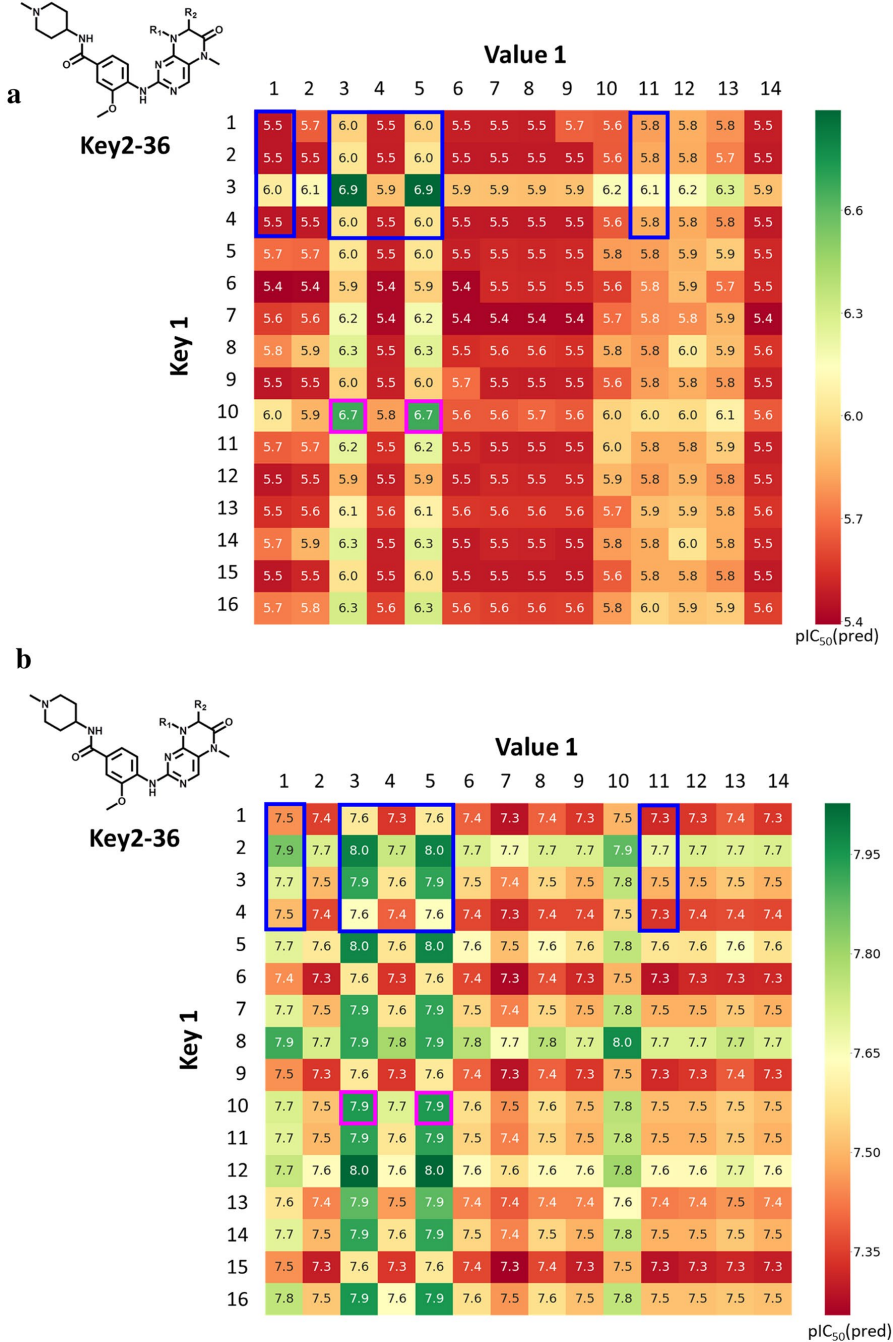
Dual-target activity prediction. In **a** and **b**, relative potency predictions are reported for virtual compounds from the expanded Key2-36 SARM (according to Fig. 3a) against BRD4 and PLK1, respectively. Compound cells are color-coded using a continuous spectrum from red (lowest potency) over yellow to green (highest). Cells containing compounds from the original SARM are framed in blue and cells containing two prioritized candidate inhibitors are framed in pink

The two models were then independently used to predict potency values for virtual compounds from expanded SARMs. Of note, since the models were derived exclusively on the basis of active compounds, all virtual compounds were predicted to be active (within the potency value range of the training set). Thus, predicted potency values must be considered on a relative scale for new compounds. Figure 5a and b report exemplary potency predictions for the expanded Key2-36 SARM (according to Fig. 3a). For known inhibitors, experimental potency values are reported. For most virtual compounds, low BRD4 potency was predicted (Fig. 5a). Only a few compounds were predicted (or known) to have highest potency values within this data set, which represented combinations of key 3 and key 10 from the original SARM and DeepSARM, respectively, and values 3 and 5 (original SARM). The compounds representing combinations of key 3 and values 3 or 5 were known dual-target ligands contained in the SARM. Encouragingly, for the two DeepSARM candidates, comparable potency was predicted.

For PLK1, the predicted potency range for virtual compounds was very narrow (Fig. 5b), reflecting the potency distribution in the original compound data set. However, the two DeepSARM candidates prioritized on the basis of BRD4 predictions were again among the compounds with highest predicted potency values, comparable to the two known dual-target inhibitors. Hence, on a relative scale, these virtual compounds were preferred candidates for BRD4/PLK1 dual-target ligands, given the consistency of their predictions.

### Candidate compounds and follow-up analysis

**Fig. 6:**
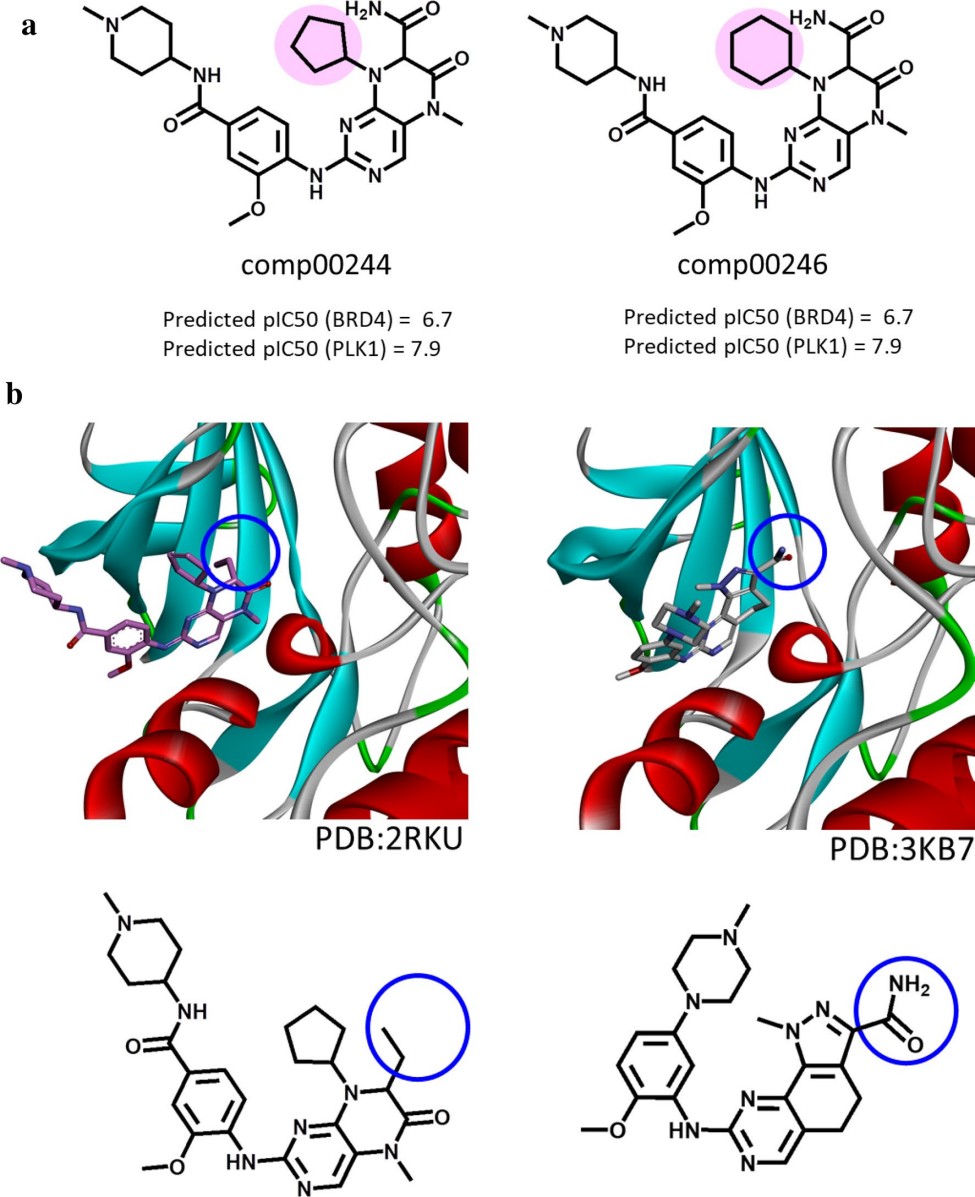
Candidate compounds and X-ray structures. **a** The structures of the two candidate compounds (indexed comp00244 and comp00246) are displayed. These virtual candidates are analogs (the distinguishing structural modification is highlighted in pink). **b** X-ray structures of closely related compounds in complex with PLK1 (ribbon representation) are shown (top). At the bottom, the structures of these inhibitors are displayed in corresponding orientation reflecting a similar binding mode. An ethyl vs. carboxamide R-group replacement is encircled in blue

On the basis of the findings discussed above, we focused our attention on the two DeepSARM candidates, depicted in Fig. 6a (indexed comp00244 and comp00246, respectively). These two virtual compounds were close structural analogs only distinguished by a cyclopentane to cyclohexane ring substitution. We have searched the Protein Data Bank [37] for X-ray structures of PLK1 and BRD4 containing these or related compounds and identified a structure of a close analog of comp00244 in complex with PLK1, shown on the left in Fig. 6b. The crystallographic inhibitor was only distinguished from comp00244 by an ethyl to carboxamide substitution. Furthermore, we identified another structure of PLK1 in complex with a closely related pyrazoloquinazoline inhibitor, as shown on the right in Fig. 6b. This inhibitor displayed a very similar binding mode compared to the comp00244 analog and contained the carboxamide group of comp00244 at the corresponding position where it interacted with PLK1 residues. Hence, on the basis of these observations, the newly generated compounds comp00244/246 are very likely to at least inhibit PLK1. Of course, their predicted dual-target ligand potential awaits experimental evaluation.

### Enhanced SARM and MGM display

**Fig. 7:**
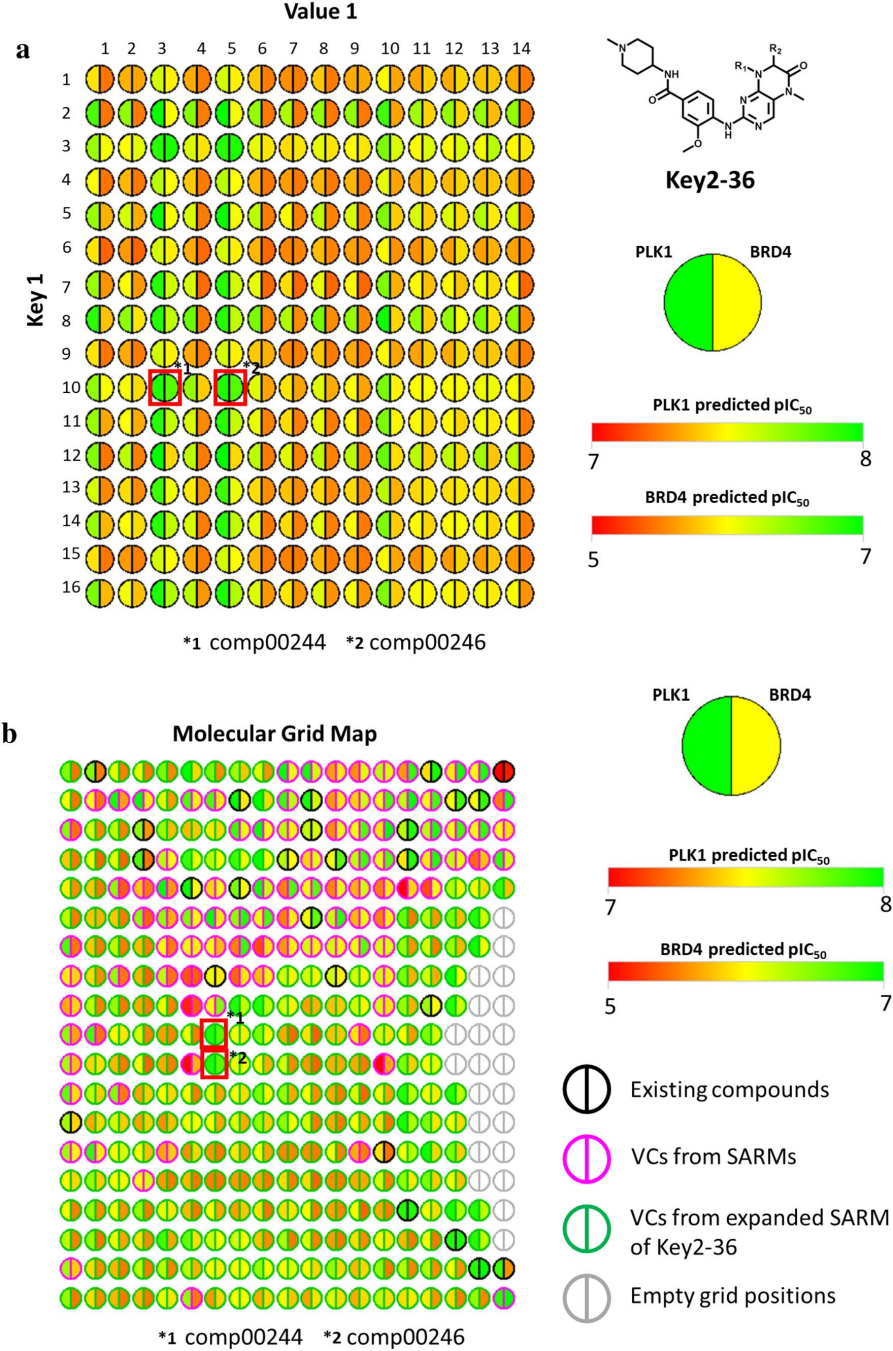
SARM and MGM display for dual-target ligand design. **a** The expanded Key2-36 SARM is shown using a modified compound representation accounting for dual-target activity. **b** The corresponding MGM representation is shown. For all compounds, potency values were predicted for both targets. The positions of virtual candidates comp00244/246 are framed in red

For dual-target ligand design, a new color-coded representation was also implemented that is applicable to both SARM and MGM. Following this design idea, compound cells in SARMs and grid positions in MGMs are represented as divided circles (nodes) color-coded by (experimental or predicted) potency values for the two targets under consideration. Figure 7a shows the expanded SARM from Fig. 5 in this intuitive dual-target view and Fig. 7b shows a corresponding MGM representation for this compound set on an (algorithmically derived) 19 × 19 grid. Here, node borders are used to distinguish different compound categories. According to the chosen color spectrum, dark green nodes represent preferred candidate compounds. Structural analogs (such as comp00244/246) occupy adjacent positions. The MGM display can be further expanded, for example, to include multiple SARMs containing dual-target ligands such that virtual candidate compounds can be viewed in context.

## Concluding discussion

Compounds with desired dual-target activity are of high interest in polypharmacology-oriented drug discovery. However, apart from dual-pharmacophore screening, computational approaches for the identification or design of dual-target ligands are still rare. The SARM approach was originally developed for different purposes. It was focused on the systematic identification and structural organization of related analog series. Furthermore, it was designed to bridge between structural analysis and compound design by extrapolating from organized series to generate new virtual analogs. Subsequently, the DeepSARM methodology was introduced to further expand analog space for given series through generative modeling, taking compound information from related targets into account. So far, DeepSARM has only been applied to compounds with activity against a single target. However, given its two-stage training scheme, we have reasoned that DeepSARM might be adapted for dual-target ligand design, which represents the concept introduced herein. The initial training phase makes it possible to focus on chemical space populated with compounds active against target groups. Subsequent fine-tuning enables the design of compounds that are likely to be active against a specific target combination from these groups. The DeepSARM approach for dual-target ligand design can be adjusted depending on the compound information that is available. In the exemplary application presented herein, we have shown that fine-tuning on the basis of only small numbers of available dual-target ligands can produce attractive candidate compounds for further exploration. DeepSARM modeling substantially extends the analog space of original SARMs, leading to SARM expansion, which also applies to dual-target ligand design, as shown herein. Candidate compounds can be selected on the basis of log_likelihood scores originating from DeepSARM and/or results from externally derived activity prediction models. Compound prioritization schemes can be modified or extended according to individual preferences. At present, dual-target ligand design via the DeepSARM framework is still at the conceptual level. However, we demonstrate the computational feasibility of the approach. The results of the exemplary application reported herein should be of sufficient interest to pave the way for other DeepSARM dual-target ligand design projects leading to experimental work. From a design perspective, a strength of the SARM data structure and its DeepSARM expansion is the visualization capacity, including MGM display, which enables intuitive access to candidate compounds, even for practicing chemists who might not be familiar with all computational details.

In conclusion, we have introduced a new concept for dual-target ligand design based upon the DeepSARM framework, which should merit further consideration. It is also hoped that our analysis might trigger further computational investigations supporting polypharmacological drug discovery.
